# Clinical value of 3D printing guide plate in core decompression plus porous bioceramics rod placement for the treatment of early osteonecrosis of the femoral head

**DOI:** 10.1186/s13018-018-0812-3

**Published:** 2018-05-30

**Authors:** Bo Li, Pengfei Lei, Hao Liu, Xiaobin Tian, Ting Wen, Ruyin Hu, Yihe Hu

**Affiliations:** 1 Department of Orthopaedics, Guizhou Provincial People’s Hospital, Guiyang, 550002 Guizhou People’s Republic of China; 2 Department of Orthopaedics, Xiangya Hospital of Central South University, Changsha, People’s Republic of China; 3 Department of Surgery, University of Pittsburgh, Pittsburgh, PA 15213 USA

**Keywords:** Femur head necrosis

## Abstract

**Background:**

The conventional method of core decompression combined with porous bioceramics rod is usually performed under C-arm fluoroscopy for the treatment of early osteonecrosis of the femoral head (ONFH). This study was to evaluate the clinical value and efficacy of three-dimensional (3D) printing guide plate in the process of core decompression plus porous bioceramics rod for the treatment of early ONFH.

**Methods:**

Forty patients were enrolled, including 20 patients undergoing the surgery with 3D printing guide plate in the experiment group and 20 controls with C-arm fluoroscopy. The following parameters such as surgery time, blood loss, fluoroscopy times, and the accuracy of core decompression for necrosis area, function outcome according to Harris Hip Score (HHS), and any possible complications were recorded and compared between the two groups. All the patients were followed up at 6, 12, and 18 months postoperatively.

**Results:**

The surgery time, fluoroscopy time, and intraoperative blood loss in the experiment group was significantly less (*P* < 0.05) than those in the control group. There was no statistical significance in the accuracy of core decompression and porous bioceramics rod placement between the two groups (*P* > 0.05). All patients were followed up for 18 months. There was a significant difference between the preoperative and final follow-up HSS scores in both groups (*P* < 0.05). In addition, there was also a significant difference between the groups in the last follow-up HSS scores (*P* < 0.05).

**Conclusions:**

Compared with the traditional method, 3D printing guide plate could shorten the surgery time and fluoroscopy times and decrease intraoperative blood loss. It seems to be an effective method in the combined core decompression with porous bioceramics rod placement for early ONFH.

## Background

Osteonecrosis of the femoral head (ONFH) is a catastrophic disease of the femoral head and affects younger patients in their thirties and fifties. ONFH has complex and poorly understood pathogenesis and, if left untreated, may seriously damage the hip joint’s function [1–3]. For ONFH within the reversible stage, core decompression of the necrotic area represents an established and commonly used technique. This therapeutical principle was developed by Ficat and Arlet in 1962 during diagnostic “functional exploration of bone” [4]. In the reversible stages, i.e., Steinberg stage I, II, and III, core decompression is supposed to reduce the intraosseous pressure and bring about reperfusion. Core decompression can be modified by additional interventions such as implantation of bone marrow cells, growth factors, or fibular grafting [5–7]. Porous biocramics beta-tricalcium phosphate is a new type of implantable biomaterial in orthopedic surgery [8, 9]. Core decompression combined with porous bioceramics rod implant is one of most effective methods in treating the early ONFH [10]. Although most surgeons can perform core decompression under a C-arm fluoroscopic imaging system, there still remains a challenge to achieve the precise location of the necrosis area and shorter operation and fluoroscopy time.

Three-dimensional (3D) printing has gradually penetrated into the field of clinical medicine [11], which may probably be used to guide the core decompression operations for the patients with ONFH. Specifically, the 3D printing technology could produce a joint surgical guide plate which can be used to preoperatively estimate the necrosis area and depth of core decompression, and mimic placing the porous bioceramics rod. Besides, based on the individualized 3D printing model, surgeons could have a better understanding of the anatomical structure and the disease, which may contribute to shorten operation time, promote surgery accuracy, and reduce complications [12]. To our knowledge, there are few reports regarding 3D printing used in the combined core decompression and porous bioceramics rod placement [13]. In this study, we aimed to assess the clinical value and efficacy of 3D printing model in the combined surgery for the treatment of ONFH.

## Methods

The study was approved by the Ethics Committee of Guizhou Provincial People’s Hospital (series number: 2017-009). Written informed consent was obtained from each subject.

### Patients and methods

Patients with early ONFH receiving the core decompression plus porous bioceramics rod placement from October 2015 to June 2016 were enrolled for this study. ONFH was diagnosed according to radiological examination and clinical evidence. Patients were preoperatively evaluated using Steinberg staging. The inclusion criteria were as follows: Steinberg stage I, II, or IIIA and consistent hip pain for at least 6 months without obvious improvement after non-operational treatment. The exclusion criteria were as follows: bone lesions but not conform to femoral head necrosis and Steinberg stage IIIB, IIIC, IV, or V. The written informed consents of all patients were obtained.

The patients were assigned into experimental group and control group according to the decision of therapeutic regimen made by the chief surgeons. Patients in the experimental group received core decompression with porous bioceramics rod placement assisted by 3D printing guide plate, while patients in the control group had the same surgery under the traditional X-ray-assisted procedure.

### Surgical procedures

**Fig. 1:**
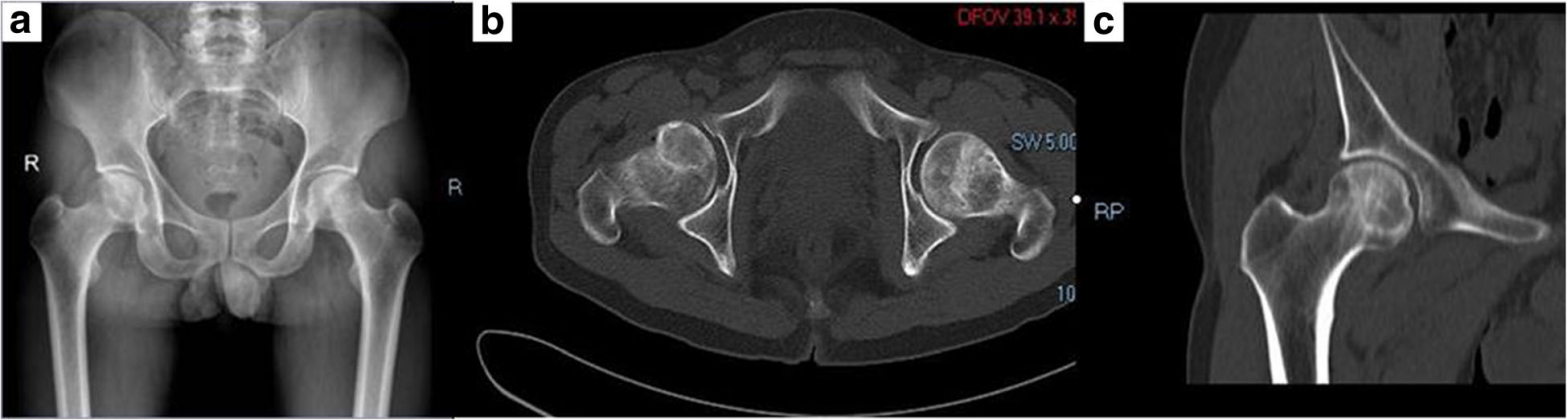
**a**–**c** Pre-operative X-ray and CT images of hip joint

**Fig. 2:**
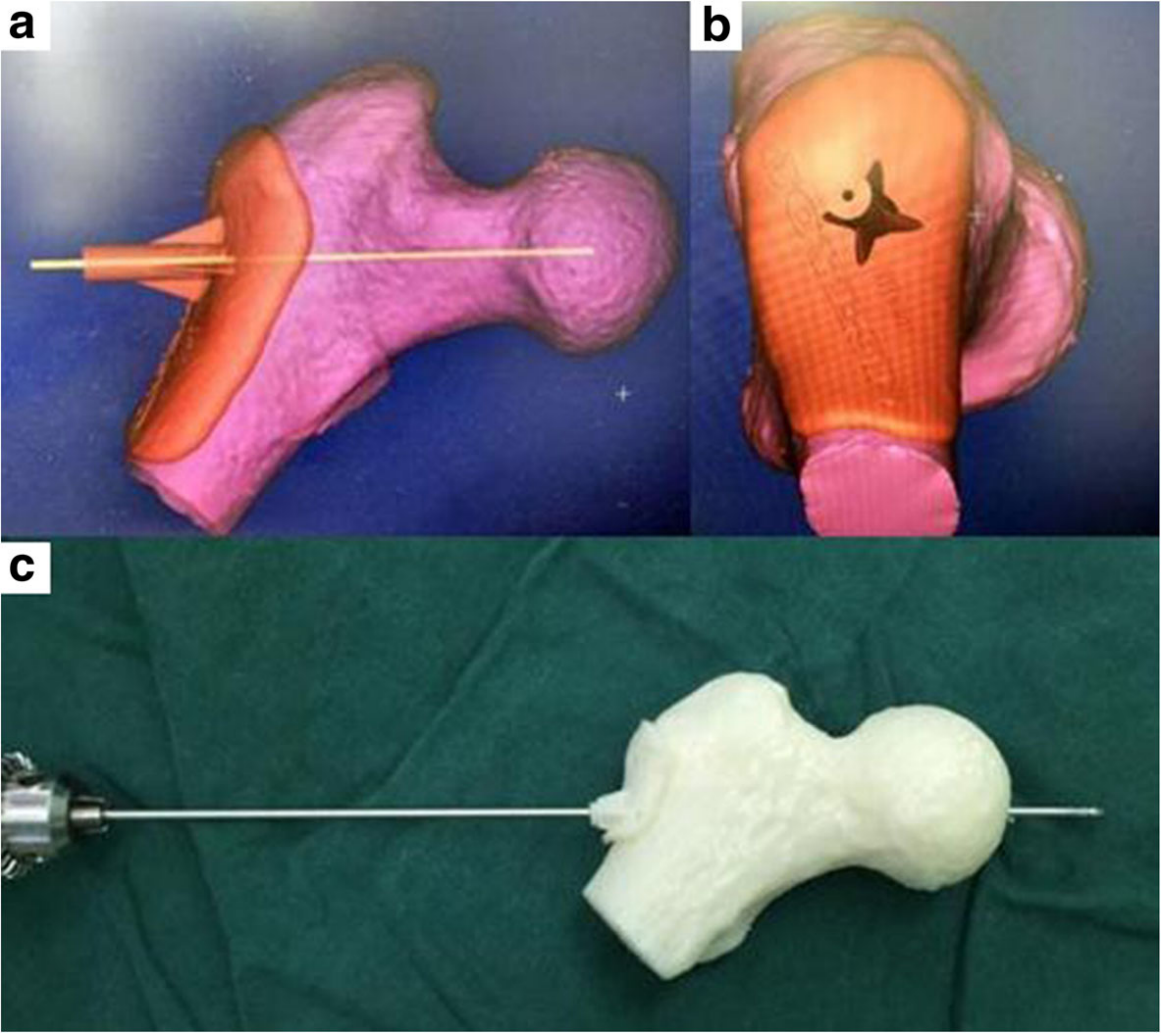
**a** The pre-operative 3D CT reconstruction images. **b** A virtual guide pin path was established on the proximal femur model using MedCAD, making the path reach to the target region. **c** The guide pin path was established with the assistance of the corresponding guide pin guide plate, which was performed at the opposite side by the same method

All patients underwent preoperative imaging examination, such as hip joint-anterior/lateral radiographs, MRI, and CT for protopathic certification (Fig. 1). In the experimental group, preoperative thin-slice CT scans of the hip joint were obtained using the Siemens 64 row spiral CT (Siemens, Munich, Germany). The scanning parameters were as follows: scanning voltage, 130 kV; scanning thickness, 0.625 mm; and the matrix, 512 × 512. Digital imaging and communications in medicine format CT data were imported into Simpleware 7.2 (Simpleware Ltd., Exeter, UK) to reconstruct the 3D hip model of stereolithography format. The experimental group received hip joint CT 3D reconstruction with a layer of 0.625 mm. The digital design was created using Geomagic (3D Systems, Valencia, CA, USA). The 3D printer was used, and the printing material was polyamide PA2200 (Fig. 2). The bio-model and its corresponding template were placed together, and a standard electric power drill was used to locate the decompression trajectory into the bio-model at the predefined site. 3D printing guide plate was sterilized before the operation.

The operation in both groups was performed in a minimally invasive lateral approach (3–4 cm) by the same surgeon. The patient was in the supine decubitus position with the affected hip draped freely. The fascia lata and the vastus lateralis muscle were split down to the lateral side of the proximal part of the femur. After full exposure, the 3D printing guide plate was tightly attached to the proximal part of the femur, and one Kirschner wire was inserted into the pinhole on the guide plate to obtain a core decompression position. Then, a guide needle was inserted from the lateral femoral cortex into the necrosis area to guide the drilling hole with a special core reamer (8–10 mm in diameter), and finally, a bony channel up to about 5 mm depth underneath the articular cartilage surface was established. The needle and reamer were withdrawn. The core decompression was done: the necrotic bone and granulation tissue were scraped thoroughly with a curette. After core decompression, a porous bioceramics rod (10 mm in diameter and 90 mm in length) purchased from Shanghai Bio-lu Biomaterials Co., Ltd., China, was inserted according to the depth of the channel. C-arm fluoroscopy was used to confirm the angle, position, and depth of core decompression. The control group received traditional X-ray-assisted core decompression and porous bioceramics rod placement.

#### Clinical evaluation and follow-up

Radiologic evaluation included standard anteroposterior and lateral X-ray and hip joint. CT examinations were performed in each patient preoperatively and 6 months, 12 months, and 18 months postoperatively. The following indexes were recorded, including the intraoperative blood loss, surgical time, fluoroscopy times, the accuracy of core decompression for necrosis area, and any possible complications in both groups. The amount of intraoperative blood loss was roughly calculated by the amount of blood in suction bottle and the weight gain of the gauze. Accuracy of core decompression was defined as the successful positioning of Kirschner wire in the necrosis area.

Outcomes were measured and recorded by Harris Hip Score (HHS) which contains four items: pain, function, activity, and motion of the hip. The HHS was categorized as excellent (90–100 points), good (80–89 points), fair (70–79 points), or poor (< 69 points).

##### Statistical analysis

Statistical analysis was performed with SPSS 19.0 Statistical software. Parametric data are presented as means ± standard deviation (SD) and compared with the independent *t* test. Non-parametric data are expressed as percentage and compared with the χ^2^ test. A *P* value of less than 0.05 is considered as significant.

A post hoc power analysis was performed using a G*Power software [14] (version 3.1; Düsseldorf University, Düsseldorf, Germany) for each sample size of the cohorts and the means (and standard deviations) of the outcome measure surgical time of each group, resulting in a power value of 0.95.

## Results

### Baseline data

A total of 40 patients were enrolled at our service between October 2015 and June 2016 regarding their eligibility for the present study. In the experimental group (11 males, 9 females; average age 42.3 ± 5.2, range of 35–50 years), there were six hips with stage I ONFH, eight hips with stage II, and six hips with stage IIIA. In the control group (14 males, 6 females; average age 39.7 ± 6.4, range of 31–48 years), there were seven hips with stage I ONFH, six stage II, and seven stage IIIA. The baseline characteristics of the participants in the groups were compared, and the results are described in Table 1. There was no statistically significant difference regarding age, sex, BMI, etiologic factors, Steinberg stage, and preoperative Harris Hip Score between the two groups (all *P* > 0.05).

**Table 1:** Patient characteristics (*n* = 40, means ± SD)

	Experiment group (*n* = 20)	Control group (*n* = 20)	*P* values
Age, years	42.30 ± 9.20	39.70 ± 8.40	0.357
Sex			0.327
Male	11	14	
Female	9	6	
BMI	24.95 ± 2.59	24.47 ± 2.05	0.152
Etiologic factors, number of hips (%)			0.414
Alcohol	10	7	
Steroids	7	11	
Idiopathic	3	2	
ARCO stage			0.803
I	6	7	
II	8	6	
IIIA	6	7	
Preoperative Harris Hip Score	72.70 ± 3.81	70.40 ± 3.36	0.051

### Surgical profiles

The surgical profiles were shown in Table 2. The surgery time (29.9 ± 2.1 min) in the experiment group was significantly less (*P* < 0.05) than that of the control group (40.5 ± 2.2 min). There was less intraoperative blood loss in the experiment group (18.0 ± 11.1 mL) than that of the control group (39.9 ± 13.0 mL) with statistical significance (*P* < 0.05). The fluoroscopy times for core decompression and porous bioceramics rod placement were 0.9 ± 0.4 in the experimental group and 4.1 ± 0.7 in the control group (*P* < 0.05), respectively.

**Table 2:** Surgical profiles in two groups

	Experiment group (*n* = 20)	Control group (*n* = 20)	*P*
Surgery time, min	29.93 ± 2.13	40.51 ± 2.24	0.001
Intraoperative blood loss, ml	18.04 ± 11.14	39.91 ± 13.03	0.001
Fluoroscopy times	0.92 ± 0.41	4.14 ± 0.73	0.001

### Surgical results and complications

**Fig. 3:**
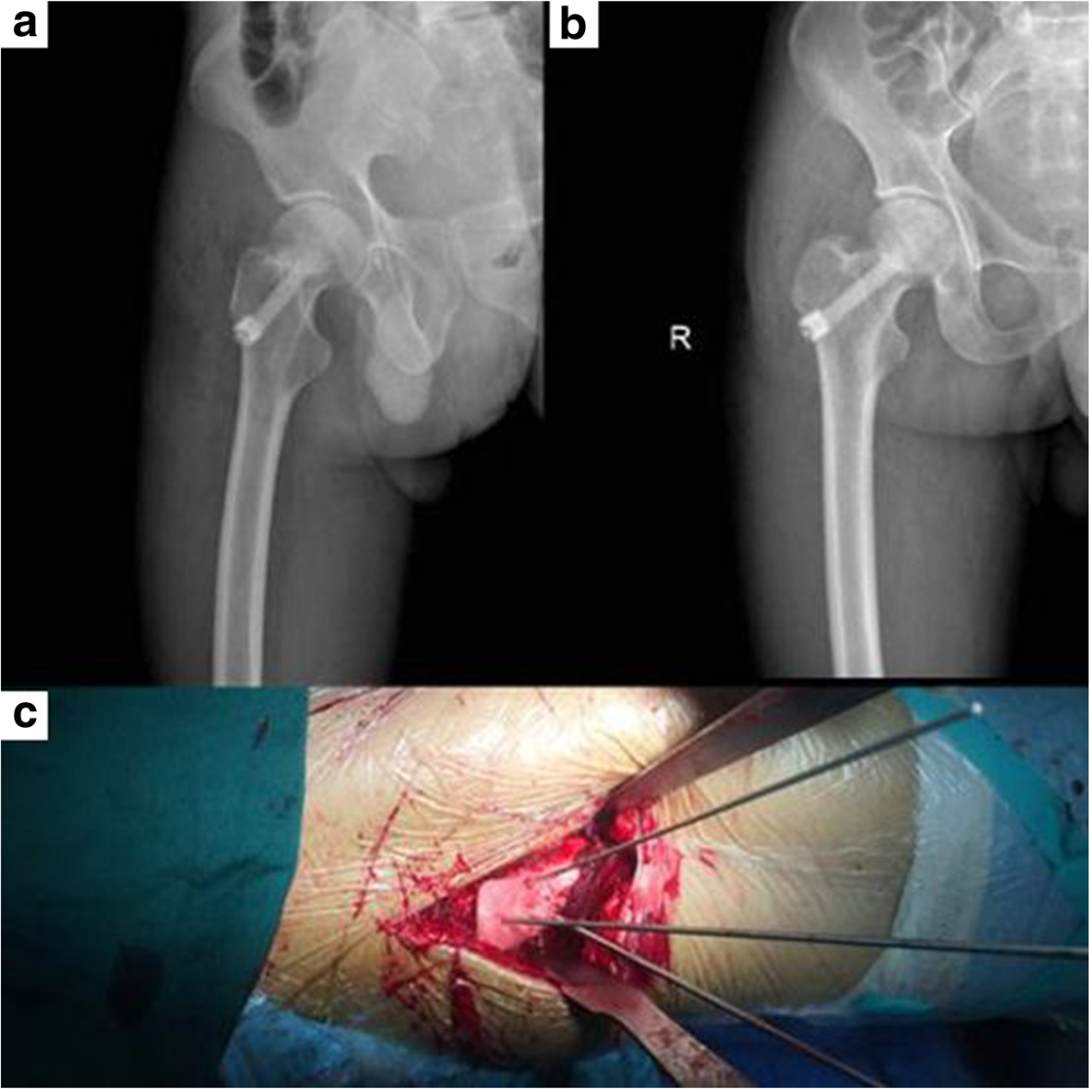
**a** The post-operative lateral X-ray films. **b** The post-operative anterior-posterior X-ray films, which showed the good position of core decompression with porous bioceramics rod. **c** The guide pin path was established under the assistance of individualized 3D guide plate during the operation

The accuracy of core decompression and porous bioceramics rod placement was good in 20 (100%) in the experimental group and 18 (90%) in the control group, with no statistical significance (*P* > 0.05). The post-operative anteroposterior and lateral X-ray film and hip joint CT showed that core decompression and porous bioceramics rod placement was satisfactory (Fig. 3). No obvious complications were observed in either group.

All patients were followed up for 18 months. The Harris scores in both groups postoperatively at different follow-up periods were statistically significantly improved in comparison to that of the preoperative values. In addition, there were statistically significant differences between the two groups regarding the postoperative Harris scores at 6, 12, and 18 months of follow-up (*P* < 0.05) (Table 3).

**Table 3:** Harris scores in two groups

	Preoperatively	6 months postoperatively	12 months postoperatively	18 months postoperatively
Experiment group (*n* = 20)	72.70 ± 3.81	88.55 ± 1.47^a,b^	91.80 ± 1.51^a,b^	94.20 ± 0.95^a,b^
Control group (*n* = 20)	70.40 ± 3.36	83.75 ± 2.23^a^	89.65 ± 1.46^a^	91.85 ± 0.99^a^
*P* values	0.051	< 0.05	< 0.05	< 0.05

^a^vs preoperative values, *P* < 0.05

^b^vs control group, *P* < 0.05

## Discussion

With the rapid progression of digital technology, the clinical applications of 3D measurement and operative simulation have gradually increased. The 3D printing technique is a modality of rapid prototyping (RP) technique based on 3D models and uses computer software and CNC forming system to produce a physical product using special materials, such as metal powder, ceramic powder, and cellular tissue. In orthopedic surgery, this technique is based on the CT scan image to rebuild the target bones including other anatomical structures. RP has demonstrated significant potential as an effective visualization tool alongside the conventional imaging methods for preoperative planning and intraoperative surgical guidance [15]. RP technique is mainly applied in dentistry and maxillofacial surgery [16]. The 3D printing individual guide plates have been reported to be successfully used in spinal surgery, joint surgery, and tumor surgery [17–19]. The 3D printed “polyamide PA 2200” plate has excellent properties of heat resistance, abrasion resistance, mechanical strength, and mechanical property. It can maintain a certain strength and is not easily deformed. During the installation of the guide plate and the operation process, the accuracy will not be affected due to the deformation of the guide plate. Several small holes on the guide plate were designed for the insertion of K-wire, so as to anchor the plate on the femur.

This study showed that 3D printing guide plates played a positive role in the combined core decompression with porous bioceramics rod for early ONFH. It shortened operation time and fluoroscopy time and decreased blood loss.

As for the accuracy of core decompression and porous bioceramics rod placement, there was no statistical significance between the two groups. This was possibly due to that most of the patients in the present study were without anatomical abnormality, and thus, the surgeon could achieve satisfactory result with his experience and the help of intraoperative fluoroscopy.

There are several advantages in 3D printing individual guide plate. Firstly, the anatomical morphology was shown in the surgical plan prior to surgery. Thus, the surgeon can have a better master of the location, angle, orientation, and the depth of the core compression. Secondly, it is simple to apply and does not request much expertise of the surgeon; the preoperatively prepared individual guide plate can be used intraoperatively to assist with surgical navigation and precise location of the instrumentation. Thirdly, core decompression could be accurately performed which avoids damaging the articular cartilage. Fourthly, in contrast to the traditional X-ray-assisted method, this technique eliminates the need for complex equipment and time-consuming procedures in the operating room; thus, it decreases the surgical duration.

The technique also has potential sources of error [20, 21]. If without sufficient thin cuts of CT scans, it may lead to an inaccurate 3D representation of the bony surface and a poorly fitting drill template. The RP model could deviate from the computer 3D model, but existing RP technology can control deviation to 0.1 mm. This technique requires more clean preparation of the bone surface than in conventional surgeries, including thorough removal of the attached muscle and soft tissue without any potential damage to the bony surface structure. In this circumstance, soft-tissue release takes certain time. However, the repeated positioning by C-arm fluoroscopy will cost much more surgical time. Thus, the 3D printing guide plate technique reduced the surgery time and fluoroscopy time relative to the C-arm fluoroscopy technique.

## Conclusions

Compared with the traditional method, 3D printing guide plate could shorten the operation time and fluoroscopy time and decrease intraoperative blood loss. It is suggested that 3D printing guide plate seems effective in the combined core decompression with porous bioceramics rod placement for early ONFH.

## Abbreviations

3D: Three-dimensional
ONFH: Osteonecrosis of the femoral head
RP: Rapid prototyping
